# Prediction of Drug-Likeness Using Deep Autoencoder Neural Networks

**DOI:** 10.3389/fgene.2018.00585

**Published:** 2018-11-27

**Authors:** Qiwan Hu, Mudong Feng, Luhua Lai, Jianfeng Pei

**Affiliations:** ^1^Center for Quantitative Biology, Academy for Advanced Interdisciplinary Studies, Peking University, Beijing, China; ^2^BNLMS, State Key Laboratory for Structural Chemistry of Unstable and Stable Species, Peking-Tsinghua Center for Life Sciences at College of Chemistry and Molecular Engineering, Peking University, Beijing, China

**Keywords:** drug-likeness, ZINC, MDDR, deep learning, auto-encoder

## Abstract

Due to diverse reasons, most drug candidates cannot eventually become marketed drugs. Developing reliable computational methods for prediction of drug-likeness of candidate compounds is of vital importance to improve the success rate of drug discovery and development. In this study, we used a fully connected neural networks (FNN) to construct drug-likeness classification models with deep autoencoder to initialize model parameters. We collected datasets of drugs (represented by ZINC World Drug), bioactive molecules (represented by MDDR and WDI), and common molecules (represented by ZINC All Purchasable and ACD). Compounds were encoded with MOLD2 two-dimensional structure descriptors. The classification accuracies of drug-like/non-drug-like model are 91.04% on WDI/ACD databases, and 91.20% on MDDR/ZINC, respectively. The performance of the models outperforms previously reported models. In addition, we develop a drug/non-drug-like model (ZINC World Drug vs. ZINC All Purchasable), which distinguishes drugs and common compounds, with a classification accuracy of 96.99%. Our work shows that by using high-latitude molecular descriptors, we can apply deep learning technology to establish state-of-the-art drug-likeness prediction models.

## Introduction

Over the past several decades, various novel and effective techniques, such as high-throughput screening(HTS), fragment-based drug discovery (FBDD), single-cell analysis, have been developed and led to remarkable progresses in the field of drug discovery. However, it is noted that the amount of new chemical entities (NCEs) approved by FDA did not grow as rapidly as expected (Darrow and Kesselheim, 2014). According to statistics, the success rate of candidate compounds found in preclinical detection is about 40%, while the rate of candidate compounds entering the market is only 10% (Lipper, 1999).

About 40% of the candidate compounds not being marketed is due to their poor biopharmaceutical properties, also commonly referred to as drug-likeness, which includes poor chemical stability, poor solubility, poor permeability and poor metabolic (Venkatesh and Lipper, 2000). Drug-likeness, derived from structures and properties of existing drugs and drug candidates, has been widely used to filter out undesirable compounds in early phases of drug discovery. The initial concept of drug-like rules is proposed by Lipinsky, known as the rule-of-five which contains four simple physicochemical parameter definitions (MWT ≤ 500, log P ≤ 5, H-bond donors ≤ 5, H-bond acceptors ≤ 10) (Lipinski, 2004). Using these definitions may predict whether a compound can become an oral drug candidate. In 2012, Hopkins et al. propose the quantitative estimate of drug-likeness (QED) measure, which was a weighted desirability function based on the statistical distribution of eight selected molecular properties for a set of 771 orally absorbed small molecule drugs and applied to molecular target druggability assessment (Bickerton et al., 2012). Due to the ambiguous definition of molecular properties between the drugs and non-drug and the prediction is not satisfactory with few descriptors, later works tried to combine more comprehensive descriptors and a large amount of compound data to develop drug-likeness prediction models with high accuracies from a quantitative perspective.

A drug-likeness prediction model introduced by Wagener et al., involved molecular descriptors related to numbers of different atom types and decision trees for discriminating between potential drugs and nondrugs. The model was trained using 10,000 compounds from the ACD and the WDI, and its prediction ACC on an independent validation data set of 177,747 compounds was 82.6% (Wagener and van Geerestein, 2000). In 2003, Byvatov and co-workers used various different descriptor sets and descriptor combinations to characterize compound and applied SVM and artificial neural network (ANN) systems to solve the drug/nondrug classification problem. Both methods reached 80% correct predictions and their results indicated SVM seemed to be more robust (Byvatov et al., 2003). A later model reported by Muller was also based on SVM with a careful model selection procedure for improving the prediction results of Byvatov et al. (2003) (Müller et al., 2005). In 2008, Li et al implemented ECFP_4 (Extended Connectivity Fingerprints) for characterizing the molecules and used a probability SVM model to classify drug-like and non-drug-like molecules. The model significantly improved the prediction ACC when compared to previous work on the same data sets, and it is surprising that when using a larger data set of 341,601 compounds the classifier increased the ACC to 92.73% (Li et al., 2007). Schneider et al. applied decision trees to perform a gradual *in silico* screening for drug-like compounds based on SMARTS strings and the molecular weight, XlogP, and the molar refractivity as descriptor space for compounds (Schneider et al., 2008). In 2012, Tian et al implemented 21 physicochemical properties and the LCFP_6 fingerprint encoding molecules and used the naive Bayesian classification (NBC) and recursive partitioning (RP) to construct drug-like/non-drug-like classifier, which achieved 90.9% ACC (Tian et al., 2012). These studies showed that machine learning techniques are highly potential for the drug-likeness prediction problem combined with big data sets.

Deep learning is a new wave of machine learning based on artificial neural networks (ANN) (Bengio, 2009; Vincent et al., 2010). Since 2006, DL has been showing superior performances in many fields, such as computer vision (Hinton et al., 2006; Coates et al., 2011; Krizhevsky et al., 2012; He et al., 2016), natural language processing (Dahl et al., 2012; Socher et al., 2012; Graves et al., 2013; Mikolov et al., 2013; Bahdanau et al., 2016), bioinformatics and chemoinformatics (Di Lena et al., 2012; Lyons et al., 2014; Heffernan et al., 2015; Chen et al., 2016; Zeng et al., 2016). Compared to traditional machine learning methods, DL with multiple levels of layers can automatically transform raw data into a suitable internal feature representation which is beneficial for detection or classification tasks (LeCun et al., 2015). In this study we used deep autoencoder neural networks to construct powerful prediction models for drug-likeness and manually built three larger data sets abstracted from MDDR (MACCS-II Drug Data Report [MDDR], 2004), WDI (Li et al., 2007), ACD (Li et al., 2007) and ZINC (Irwin et al., 2012; Sterling and Irwin, 2015). The molecular descriptors of compound were calculated by Mold2 (Hong et al., 2008) and Padel (Yap, 2011). The classification accuracies of drug-like/non-drug-like model are 91.04% on WDI / ACD databases, and 91.20% on MDDR /ZINC, respectively. The performance of the models outperforms previously reported models. In addition, we developed a drug/non-drug-like model (ZINC World Drug vs. MDDR), which distinguishes drugs and common compounds, with a classification ACC of 96.99%. Our work shows that by using high-latitude molecular descriptors, we can apply DL technology to establish state-of-the-art drug-likeness prediction models.

### Datasets

#### Benchmark Datasets

In this study, the whole chemical space was divided into drug, drug-like and non-drug-like. Marketed drug molecules were represented by ZINC WORLD DRUG (Sterling and Irwin, 2015) (version 2015, 2500 molecules) dataset. Drug-like molecules were represented by MDDR (MACCS-II Drug Data Report [MDDR], 2004) (200 k molecules) dataset and WDI (Li et al., 2007) (version 2002, 40k molecules) dataset. Non-drug-like molecules were represented by ACD (Li et al., 2007) (version 2002, 300 k molecules) and ZINC ALL PURCHASABLE (Irwin et al., 2012) (version 2012) datasets; the latter was randomly sampled to reduce its size to 200 k. Originally, drug-like datasets contained both marketed and drug-like molecules, and non-drug-like datasets contained the other two datasets. All datasets contained 2D molecular structure information in SDF format. Detailed information of the dataset pairs used in this study can be found in Table 1.

**Table 1:** Detailed information of the dataset pairs.

Dataset pair	Number of positive	Number of negative	Total
WDI/ACD	38,260	288,540	326,800
MDDR/ZINC	171,850	199,220	371,070
WORLDDRUG/ZINC	3,380	199,220	202,600

#### Data Preprocessing

Data cleaning can be a crucial step in cheminformatics calculation, as expounded by Fourches et al. (2010). We used a process (see Table 2) similar to that of Fourches et al. to preprocess our raw data downloaded, making it less error-prone in descriptor calculation. After descriptor calculations, we also post-processed the resulting descriptor matrix (see Table 2).

**Table 2:** Data preprocessing and post-processing steps used in this study.

Data processing	
Step Name/Software	Step description
Element filter/KNIME (Berthold et al., 2009)	Hydrocarbons are removed. Molecules containing elements other than C H O N P S Cl Br I Si are removed.
Remove Mixture/KNIME (Berthold et al., 2009)	All records containing more than one molecules are removed.
Standardize/ChemAxon Standardizer (ChemAxon Standardizer, 2010)	Neutralize, tautomerize, aromatize, and clean 2D
Remove duplicate/OpenBabel (O’Boyle et al., 2011)	Two molecules having the same InChI(including stereochemistry) means duplication. If a molecule appears in both drug set and nondrug set, it is removed from nondrug set. As for duplications in the same set, only the one that appears first is kept.
Data post-processing	
Remove error values/Python	If a descriptor has the value of N/A or ‘infinity’, the molecule it belongs to is removed.
Remove constant descriptors/Python	If a descriptor has the same value across all molecules, the descriptor is removed from the descriptor list.

#### Descriptor Calculation

We used 2D descriptors to encode the molecules. Molecules after preprocessing were calculated by MOLD2 (Hong et al., 2008), resulting a descriptor matrix of ∼700 descriptors per molecule. Then descriptor matrix was subjected to post-processing described in Table 2. We also tried the Padel descriptors (Yap, 2011), which showed inferior performance in this study and was discarded.

#### Over-Sampling Algorithms

Due to the special classification task, the positive and negative samples collected by us were not balanced in this study. Predictive model developed using imbalanced data could be biased and inaccurate. Therefore, we adopted two methods to balance our data sets to make the ratio of positive and negative samples approximately equal. The first method was to copy the minority class making the ratio 1:1, the second one was to use SMOTE (Chawla et al., 2002; Han et al., 2005; Nguyen et al., 2011), which is an improved scheme based on random oversampling algorithm. Here we used imbalanced-learn package downloaded from^1^ to apply SMOTE. For each task, we used these two oversampling methods to balance the data. For each model, firstly, we randomly split the datasets on the proportion of 9:1 as training set and validation set, secondly, we used the above two methods to balance the training set, so that the number of positive and negative samples during training was equal. The training set was used to train models with 5-CV and the additional validation set was used to evaluate models.

## Materials and Methods

### Stacked Autoencoder

**FIGURE 1:**
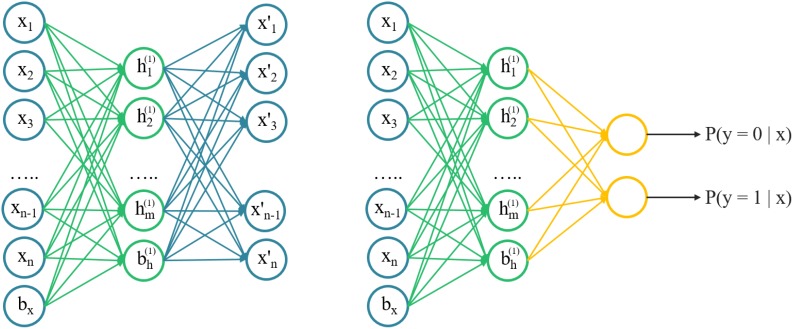
A schematic architecture of a stacked autoencoder. Left) the architecture of autoencoder, layer-by-layer can be stacked. Right) a pre-trained autoencoder to initialize a fully connected network with the same structure for classifying.

An autoencoder was an unsupervised learning algorithm that trains a neural network to reconstruct its input and more capable of catching the intrinsic structures of input data, instead of just memorizing. Intuitively, it attempted to build an encoding-decoding process so that the output $\hat{x}$ of the model is approximately similar to the input x. The SAE was a neural network consisting of multiple layers of sparse autoencoders, where the output of each layer was connected to the inputs of the successive layer. A schematic architecture of a SAE was shown in Figure 1. We trained the AE model with 2D chemical descriptors to find the intrinsic relationship between descriptors, then used the parameters of the AE model to initialize the classification model.

### Defining Models

According to the partition of chemical space into drug, drug-like and non-drug-like, there can be two kinds of classification models, drug-like/non-drug-like, drug/non-drug-like. The first one matched the traditional definition of drug-likeness. The second one also bore considerable practical value, but no model had been published to address it. In this study, to address drug-like/non-drug-like classification, we proposed two models, MDDRWDI/ZINC (which means MDDR and WDI as positive set, ZINC as negative set) and WDI/ACD. To address drug/non-drug-like classification, we proposed WORLDDRUG/ZINC (which means ZINC WORLD DRUG as positive set, ZINC ALL PURCHASABLE as negative set) model.

### Network Training and Hyperparameter Optimization

In this study, we used the open-source software library Keras (Chollet, 2015) based on Tensorflow (Abadi et al., 2016) to construct SAE model and classification model. Firstly, a single hidden layer AE was trained. The number of hidden layer nodes K, was a hyperparameter needs to be compared across different networks and tuned. During training, we used Truncated-Normal initializer to generates a truncated normal distribution of layer weights. In all case, we applied Bayesian optimization (Hyperas, a python library based on hyperopt^2^) to optimize the hyperparameter, such as the number of hidden layer nodes K, the value of L2 weight regularizer, the value of dropout, the type of activation function, the type of optimizer, the value of batch size. The final optimal hyper-parameter settings were listed in Table 3.

**Table 3:** Hyper-parameter settings of the stacked autoencoder.

Hyperparameter	Setting
Initializer	TruncatedNormal
Number of hidden layers	1
Number of hidden layer nodes	512
L2 Normalization term	1e-4
Dropout rate	0.14
Activation	Relu
Batch size	128
Optimizer	Adam
Loss	mse for AE, binary crossentropy for classifier

Considering that although the data set has been balanced, the model results may be overfitting, so we optimized the weight of the positive and negative sample loss of the logarithmic likelihood loss function as:

(1) $$L=-\sum_{k=1}^{n}\left(wy_{k}(log\;a_{k})+(1-w)(1-y_{k})log(1-a_{k})\right)$$

where y_k_ represented the k^th^ compound label. y_k_ = 1 or 0, means k^th^ compound was the drug-like or non-drug-like compound, respectively. a_k_ = P(y_k_ = 1|x_k_) was the probability to be the drug-like compound of k^th^ compound calculated by model. w was the weight of the positive sample loss. For different cases, we chose the most suitable w from the range of (0.5∼1.0) to avoid overfitting. Then we trained all models with 5-CV and enforced early stopping based on classification ACC on the test set. Finally, each case had 5 trained models and the average value was the final judgement of these models.

### Model Evaluation

All models were evaluated by five indexes. The ACC, SP, and sensitivity(SE), MCC, area under the receiver operating characteristic curve (AUC), the previous four criteria were defined, respectively, as follow:

(2) $$ACC=\frac{TP+TN}{TP+TN+FP+FN}$$

(3) $$SP=\frac{TN}{TN+FP}$$

(4) $$SE=\frac{TN}{TP+FN}$$

(5) $$MCC=\frac{TP\times TN-FP\times FN}{\sqrt{\left(FP+TN)(FP+TP)(FN+TN)(FN+TP\right)}}$$

## Results

### Compare Different Over-Sampling Methods

After we tried pre-training on validation test with 5-CV, we found that more layers and neuron numbers did not improve the predictive power. In all case, one hidden layer was sufficient for our classification objective. By analyzing the two different over-sampling methods to balance datasets, copy the minority class and SMOTE, we found the latter can achieve better prediction accuracies in Table 4.

**Table 4:** Performance on the training sets with 5-CV.

Model	Copy the minority class				SMOTE over-sampling			
	ACC	SE	SP	AUC	ACC	SE	SP	AUC
WDI/ACD	0.8923	0.8991	0.8859	0.9598	0.9265	0.9244	0.9286	0.9783
MDDR/ZINC	0.9095	0.8855	0.9302	0.9701	0.9116	0.9141	0.9092	0.9719
WORLD/ZINC	0.9910	0.9961	0.9859	0.9986	0.9906	0.9937	0.9874	0.9990

With the same dataset, the ACC of a SVM model built by Li et al was 92.73% (Li et al., 2007) and our WDI/ACD model achieves an ACC of 92.65%, almost identical to Li’s results. Our MDDRWDI/ZINC model classified drug-like/non-drug-like molecules with a satisfactory ACC of 91.16%, making it the state-of-the-art drug-likeness prediction model. These results suggest that autoencoder is a potential machine learning algorithm in drug-likeness prediction. The ACC of our drug/non-drug-like prediction model based on World Drug/ZINC dataset was as high as 99.06%, showing that it is easier to distinguish compounds from drugs or non-drugs. Although it is not excluded that the ACC of the latter models is related to the serious imbalance of the original data set, we believe that such drug/non-drug-like prediction model will likely benefit drug development.

### Optimize the Weights in the Loss Function

We observed that when using the independent external validation set pre-segmented from the original data to evaluate model, the prediction ACC of the model tended to be slightly lower than that of training, but the sensitivity value was significantly lower and the SP value was higher (Table 5), indicating that the models have some over-fitting in training.

**Table 5:** Performance of the models on the validation sets.

Model	Using SMOTE over-sampling				
	ACC	SE	SP	MCC	AUC
WDI/ACD	0.9014	0.7683	0.9191	0.6014	0.9271
MDDR/ZINC	0.9025	0.9012	0.9036	0.8043	0.9669
WORLD/ZINC	0.9800	0.7544	0.9838	0.5690	0.9707

The underlying reason may be that the positive sample ratio in the original data was too low, and we randomly divided the positive and negative samples in the original data set according to 9:1 to build the training set and the validation set. Even if the SMOTE method was used to balance the positive and negative samples in the train set, the new positive sample generated by SMOTE depended on positive sample in the original training set, so the positive sample information of the external verification set was less included.

**FIGURE 2:**
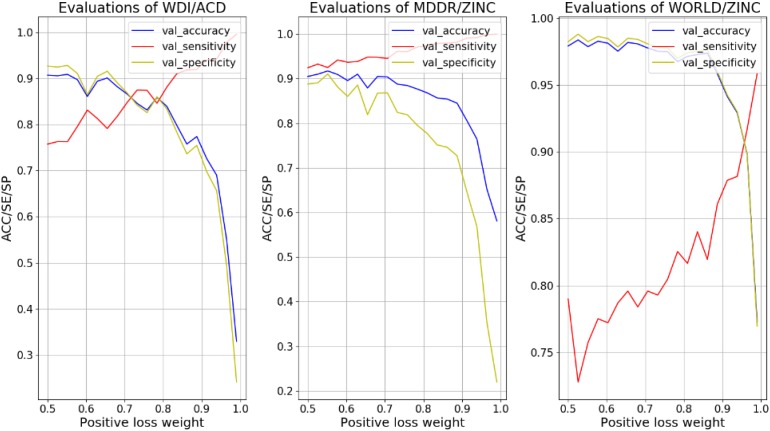
Evaluations of different models vary with weight of positive sample loss.

In order to overcome the over-fitting on the negative samples, we increased the weight of positive sample loss in the loss function to enhance the learning ability of the model to the positive sample side. We tested the weigh values (details in Formula 1) from 0.5 to 1 with 20 intervals, and record the values of ACC, SE, and SP on the validation set varying with weight, as shown in Figure 2.

For different models, the intersection point of SE and SP in the curves of Figure 2 corresponded to a balanced weight value. By fine-tuning, the weights corresponding to the four models are (0.69, 0.55 and 0.9). After using these weights for the loss functions, the ACC of the training set in different models fells slightly and the SE improves. As the model reinforces the prediction of positive samples, the SE and SP of the validation set in different models are close (shown in Tables 6, 7).

**Table 6:** Performance on the training set after optimizing the weight of loss function.

Model	SMOTE over-sampling				
	ACC	SE	SP	MCC	AUC
WDI/ACD	0.9104	0.9694	0.8515	0.8270	0.9757
MDDR/ZINC	0.9120	0.9219	0.9020	0.8243	0.9726
WORLD/ZINC	0.9699	0.9985	0.9414	0.9416	0.9955

**Table 7:** Performance on the validation set after optimizing the weight of loss function.

Model	SMOTE over-sampling				
	ACC	SE	SP	MCC	AUC
WDI/ACD	0.8458	0.8524	0.8449	0.5286	0.9253
MDDR/ZINC	0.9046	0.9174	0.8935	0.8095	0.9699
WORLD/ZINC	0.9366	0.8804	0.9376	0.4049	0.9622

Although the MCC is generally regarded as a balanced measure, it is seriously affected by the number gap between positive and negative samples of data sets and the confusion matrix calculated by the model. The MCC is satisfactory for the balanced training sets. But in the validation sets, the data set becomes more unbalanced, and the MCC becomes smaller, which was inevitable.

## Discussion

In image recognition problems, where AE was originated, several layers of AE are often stacked to make a SAE. Though SAE was found to be more powerful than single layer AE there, we found that SAE is flawed here in drug-likeness problems, making multi-layer SAE perform much poorer than single layer AE.

When a layer of AE is trained, it is expected to give output as close as possible to its input, and the error can be defined as the mean value of output minus input. In this study, when training the model, we found that the ACC of the normalized (z-score) input was much higher than scaling input to [-1,1]. After standardizing the data, the error of AE is 0.8, an order of magnitude higher than typical values in image recognition. Stacking layers of AE will further amplify the error, making the SAE-initialized NN perform poorly in classification.

We propose that such a flaw of AE stems from how input data in different dimensions are interrelated. In image recognition, each pixel is a dimension; in drug-likeness prediction and related areas, each descriptor is a dimension. The training goal of AE is to learn the relationship among dimensions, to encode input information into hidden layer dimensions. So it is very likely that AE would do worse if the relationship among dimensions is intrinsically more chaotic and irregular. The relationship among pixels is regular in that they are organized as a 2D grid and that neighbor pixels bare some similarity and complementarity. Such good properties are absent in relationship among descriptors, resulting in the failure of AE input reconstruction process. Despite the fact that AE reconstruction error is large, our model still performs well in classification. In our opinion, this is due to the regularization effect of AE pre-training. With unsupervised pre-training, the model is more capable of truly learning data, less prone to simply memorizing data.

Imbalanced data sets are a common problem. Although there are some methods such as SMOTE, which can generate new data to balance the data set, this method of generating data is much dependent on the distribution of samples. Once the distribution of samples is very sparse, then the new data is likely to deviate from the space where the original data is exited. Developing method to find data mapping spaces based on the distribution of existing data is critical to generating data to balance the data set, such as the current popular deep generation model. Developing new algorithms to train unbalanced data sets is also an important research direction.

In this study, DL has once again shown its capacity for improving prediction models. Despite the success, we believe that there is still much space for further development. A key aspect is to adapt current DL methods to specific problems. Such adaptations should be based on a better comprehension of current DL methods. That is, knowing which part of the method can be universally applied, and which part should be modified according to the nature of data. For example, in this study, we believe that the regularization effect of AE pre-training is a universal part, while the part of AE input reconstruction should be canceled or modified when input data is irregular.

## Conclusion

In this study, we manually built two larger data sets, drug-like/non-drug-like and drug/non-drug-like. Then using the AE pre-training method, we developed drug-likeness prediction models. The ACC of classification based on WDI and ACD databases was improved to 91.04%. Our model achieved classification ACC of 91.20% on MDDRWDI/ZINC dataset, making it the state-of-the-art drug-likeness prediction model, showing the predictive power of DL model outperforms traditional machine learning methods. In addition, we developed a drug/non-drug-like model (ZINC World Drug vs. ZINC All Purchasable), which distinguished drugs and common compounds, with a classification ACC of 96.99%. We proposed that AE pre-training served as a better regularization method in this study. The fail of multi-layer SAE reconstruction in this study indicated that due to the specific nature of data, some modifications may be needed when applying DL to different fields. We hope machine learning researchers and chemists collaborate closely to solve such a problem in the future, bringing further comprehension and applications of DL method in chemical problems.

## Author Contributions

QH and MF wrote the codes and analyzed the data. LL and JP conceived the work. All authors wrote the paper.

## Conflict of Interest Statement

The authors declare that the research was conducted in the absence of any commercial or financial relationships that could be construed as a potential conflict of interest.

## Footnotes

1 http://contrib.scikit-learn.org/imbalanced-learn/stable/install.html

2 https://github.com/maxpumperla/hyperas

## Abbreviations

5-CV: 5-fold cross-validation
ACC: accuracy
AE: autoencoder
AUC: areas under the receiver operating characteristic curve
DL: deep learning
DNN: deep neural network
FNN: fully connected neural network
MCC: Matthews correlation coefficient
SAE: stacked autoencoder
SE: sensitivity
SP: specificity
SVM: support vector machine.
